# Local Interactions and Dynamics in Aqueous Imidazole Probed by Vibrational and NMR Spectroscopy

**DOI:** 10.1021/acs.jpcb.6c02320

**Published:** 2026-07-20

**Authors:** Nicole Abdou, Eva Dahlqvist, Anna Martinelli

**Affiliations:** Department of Chemistry and Chemical Engineering, 11248 Chalmers University of Technology, SE-412 96 Gothenburg, Sweden

## Abstract

Imidazole is a small molecule with fundamental importance in biology, chemistry, and technology. It is found as a building unit in larger chemical systems, like amino acids, protic ionic liquids, and modern covalent organic frameworks. Water is an even more common substance of at least equal importance, not least as the charge carrier in proton conducting materials. Aqueous imidazole is a peculiar liquid system whose local structure, governed by a network of hydrogen bonding, has attracted the attention of several researchers. In this experimental work, multiple techniques, mainly vibrational and NMR spectroscopy, have been used to study aqueous imidazole solutions with the mole fraction of imidazole varying from zero to unity. Local interactions and molecular dynamics are investigated, providing results that complement previous knowledge of this molecular system. Distinct changes in the Raman spectra mark the composition with a 1:1 water/imidazole ratio as the one beyond which imidazole is solvated, whereby the local structure of crystalline imidazole is disrupted. Upon further dilution in water, the local structure changes marginally, as revealed by a careful peak fit analysis and in agreement with previous results from computational methods. Also, our experimental results help discriminate between two computational models previously used, suggesting that hydrogen bonded chains of imidazole are more frequent than other dimer forms.

## Graphical abstract

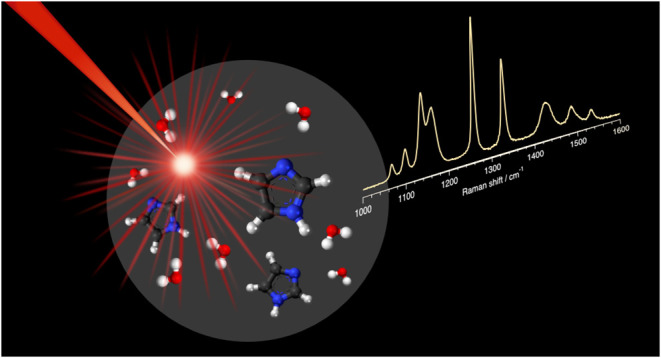

## 1 Introduction

Understanding the interaction between water and imidazole is quintessential for several biological, chemical, and technological processes. Nevertheless, a search in the literature shows that investigations of hydrated imidazole, particularly highly concentrated aqueous imidazole, are rare. The available studies have appropriately focused on understanding the formation of hydrogen bonds between these two amphoteric molecules; however, these are primarily based on computational methods with limited experimental validation.

Both water and imidazole are archetypical molecules for the conduction of protons, which relies on the formation of protonic defects within a dynamic and hydrogen-bonded structure created by self-association. Both vehicular and Grotthuss mechanisms[Fn fn1] occur in the liquid state of these molecules, with relative contributions that depend on temperature and pressure.^3–6^ In the liquid states of acidified water and imidazole, proton conduction follows the same mechanism with a similar dependence on temperature and acid concentration.^3,5^ This similarity can reasonably be translated to the case of hydrated or imidazole-doped perfluorosulfonic polymers. Understanding the short- and long-range proton motion in this type of system is a notoriously complex task, which requires deep fundamental knowledge, advanced experimental work, ideally complemented by computational insight.^4,6–10^

Notably, the synergy between the amphoteric molecules water and imidazole has been shown to be fundamental for diverse chemical processes, leading to the development of innovative applications. Key examples include the electrochemical reduction of CO_2_ in aqueous imidazole media,^11^ the utilization of water-imidazole hydrogen bonds in break-junction experiments,^12^ as well as the facilitation of proton conduction in a solid-state salt hydrate^13^ and in covalent organic frameworks.^14^

In the technological context of proton exchange membrane (PEM) fuel cells, water serves as the charge carrier, transporting protons (H^+^) from the anode to the cathode. Evaporation of water, however, causes dehydration of the membrane, limiting the operational temperature of PEM fuel cells to about 65–85 °C. Imidazole, on the other hand, is often found in the backbone of basic polymers, such as polybenzimidazole, that upon impregnation with a strong acid can be used for proton transfer in intermediate temperature fuel cells,^15^ typically operating around 160 °C. In recent years, alternative and more sustainable polymer architectures have been explored, such as membranes derived from lignin or cellulose, supposed to operate as proton conducting materials in synergy with the properties of either water or imidazole.^16,17^

Despite the rich literature dedicated to water and imidazole individually, both as bulk materials or as part of a solid or gel-like matrix, there is limited knowledge of the transport properties of their mixtures, and only a few works have investigated the local structure of such mixtures, and then mainly as highly concentrated aqueous solutions of imidazole. Moreover, the available studies have primarily used computational methods,^18–21^ with few experimental results for a direct validation.

The most relevant studies focused on elucidating the local structure and interactions in aqueous imidazole have used DFT calculations,^18^ molecular dynamic simulations^19–21^ and X-ray scattering or absorption methods.^22–24^ Computational studies performed by improved simulation models[Fn fn2] revealed that the self-association of imidazole occurs already in diluted aqueous solutions, with the intrinsic nature of water-imidazole interactions not changing dramatically with increasing content of imidazole.^19^ Also, imidazole dimers have been detected that can exist as hydrogen bonded chains, T-shaped, or H-π bonded (stacked) species, although the relative amount of each species remains unclear.^19,21^ Intriguingly, it was also found that water-imidazole interactions, rather than imidazole-imidazole interactions, are favored even as the imidazole concentration increases.^19^ These computational studies emphasized the importance of the unprotonated N^3^ site in imidazole for the interaction with water through hydrogen bonds. Complementary and very insightful results were achieved by combining X-ray scattering methods with refined DFT calculations, with the aim to understand the nucleation process in imidazole starting from supersaturated aqueous solutions.^23,24^ Together, these works confirmed strong interactions between water and imidazole, with water molecules forming linear hydrogen bonds at the N^1^H site while being more dispersed in space around the N^3^ site of imidazole. Importantly, this configuration is found to remain almost identical across different concentrations, with water-imidazole interactions dominating at all compositions. From these studies, it was concluded that imidazole minimally disrupts the hydrogen bond network of water,^24^ although further details on this aspect could not be disclosed due to the lack of experimentally recorded infrared spectra. In this broader context, the question whether water prefers to donate or accept a hydrogen bond in proximity to imidazole has attracted the curiosity of several researchers.^28,29^

With the aim of filling these knowledge gaps, e.g., on the effect of imidazole on water–water interactions and on the transport properties of aqueous imidazole solutions, this work presents a detailed analysis of vibrational (Raman and infrared) and NMR spectra, experimentally recorded for aqueous solutions of imidazole in a wide concentration range (0–8 M). More specifically, vibrational spectroscopy, combining Raman and infrared, is used to disclose structural information. Raman, infrared, and NMR spectroscopy together give valuable insights into intermolecular interactions, while pulsed field gradient (PFG) NMR is used to estimate the diffusivity of molecular species. The results presented here complement those reported in previous works, providing new pieces of information on local interactions by revisiting the assignment of key vibrational modes and by thoroughly analyzing how they change upon formation of new water-imidazole interactions. Frequency shifts, hence changes in bond length, and line broadening are analyzed by a careful peak fitting procedure and are discussed in the light of previous knowledge.

## 2 Experimental Section

### 2.1 Materials

Imidazole was purchased from Merck. The 0.6 mL ampules of DMSO-*d*_6_ 99.9% D with 0.03% TMS (v/v), used for the ^1^H NMR measurements, were purchased from Sigma-Aldrich. All chemicals were stored in a standard chemical storage cabinet and used as received. H_2_O:imidazole samples were prepared by mixing Milli-Q water (resistivity = 18.2 MΩ·cm at room temperature) and imidazole at different molar ratios, χ, with χ varying in the range 0.0 ≤ χ ≤ 1.0.

### 2.2 Raman Spectroscopy

Raman spectra were collected at room temperature, keeping the samples inside sealed NMR tubes. An InVia Reflex Raman spectrometer from Renishaw was used, using a near-infrared diode laser emitting at 785 nm as excitation and a 1200 l/mm grating, which together provide a spectral resolution of about 2 cm^–1^. The spectra were collected covering the wavenumber range 200–4000 cm^–1^, setting the laser power to ca. 9 mW at the sample and accumulating over 10 s twice. The Raman spectra are shown after subtraction of the Raman spectrum collected from an NMR tube containing neat, bulk water. Spectra were analyzed by peak fitting using the MultiPeak Fitting function embedded in the IGOR 64 software provided by WaveMetrics. The fitting model was based on a linear baseline and Lorentzian functions, whose position, width, and intensity were left as free fit parameters.

### 2.3 Infrared Spectroscopy

Infrared spectra were collected at room temperature using a Frontier MIR/FIR Spectrometer in the ATR (Attenuated Total Reflectance) mode, equipped with a single-point reflection GLADIATR diamond crystal from Pike Tech. The measured spectral range was set to be from 400 to 4000 cm^–1^, 64 scans were collected for each measurement with a spectral resolution of 2 cm^–1^. A background scan was collected and subtracted from the measured sample spectra. The infrared spectra discussed are presented without any further treatment.

### 2.4 Nuclear Magnetic Resonance (NMR) Spectroscopy

Quantitative ^1^H NMR data were collected using a Bruker Avance III HD 800 MHz spectrometer. The samples were loaded in 3 mm NMR tubes fitted with a capillary tube filled with dimethyl sulfoxide-*d*_6_ (99.9% D) with 0.03 vol % tetramethylsilane (TMS). The spectra were acquired at 298 K. The pulse angle was set to 30°, the relaxation delay was set to 15 s, and 64 scans were collected.

Diffusometry NMR measurements were conducted at 298 K on an AVANCE III HD Bruker NMR spectrometer operating at 14.1 T, equipped with a Diff30 probe, a 1*H*/2*H* double coil and a 60 A gradient amplifier. 5 mm NMR tubes were used and filled with ∼2 mL (2 cm) of sample. A standard stimulated echo pulse sequence (diffste), 18.4 μs 90-degree pulse, 1.8 s acquisition time, 3 s recycling delay, 100 ms diffusion delay (Δ), 1 ms gradient pulse duration (δ) and 100 G/cm maximum gradient strength (g) were used. The peak around 4.7 ppm was considered for the H_2_O intensity attenuation and the peak around 6.2 ppm (C^4,5^H) was considered for the imidazole. The gradient strength was calibrated against the ^1^HDO trace signal in D_2_O reference sample. The temperature was calibrated to the chemical shift difference in pure methanol^30^ prior to and after the experiments.

### 2.5 Calorimetry

DSC measurements were performed using a Mettler Toledo DSC5+ instrument equipped with an autosampler, a gas controller GC DT2 and a liquid nitrogen cooling system. For χ = 0.0, 0.1, 0.2, 0.3, and 1.0, 5 mg of sample was weighed and placed in a 40 μL hermetic aluminum crucible. For χ = 0.4 and 0.6, the appropriate amounts of imidazole and Milli-Q H_2_O were mixed directly inside a medium-pressure crucible to yield 10 mg of sample. DSC data were collected under nitrogen flow between −60 and 60 °C, except for pure imidazole, which was analyzed from −60 to 100 °C, with heating and cooling rates of 2 °C/min. Two scans were recorded for each measurement; only data from the second heating scan are reported and discussed.

## 3 Results and Discussion

The Raman and infrared spectra of an aqueous solution of imidazole with χ equal to 0.1 are shown in the common plot of Figure 1. The two spectra reveal the same set of vibrational modes, though with different intensities as a consequence of the character of the bond underlying each vibration.[Fn fn3] The (ATR) infrared spectrum shows the broad O–H bending mode of water at ca. 1637 cm^–1^, which is invisible in the Raman spectrum. All observed vibrational modes are congruent with those found in previous studies of imidazole^31–37^ and have been assigned using the results reported by Majoube et al.^35^ Table 1 summarizes the proposed assignment, which is considered robust since it results from a number of studies comparing different *ab initio* force fields, including ^15^N shift effects. For ease of interpretation, the molecular structure and atom labeling of imidazole, as well as its protonated form imidazolium, are also shown in Figure 1.

**Figure 1:**
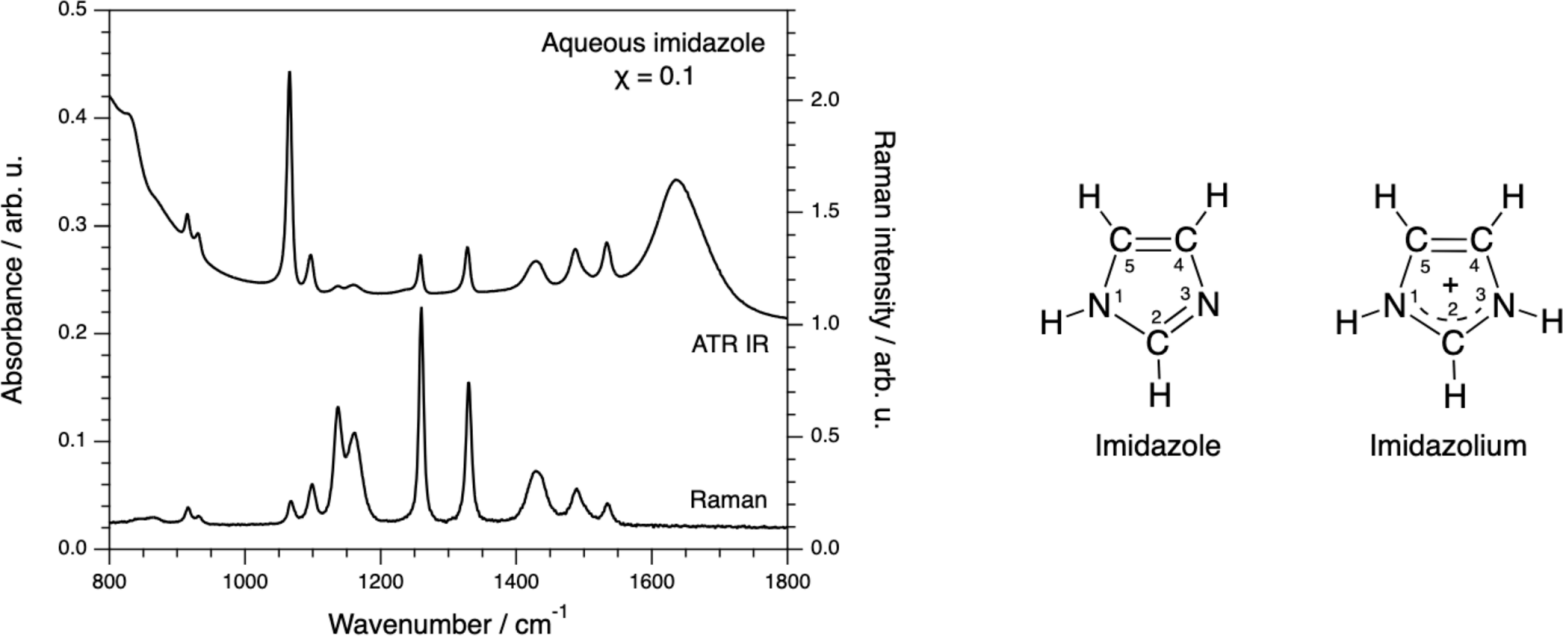
Vibrational spectra of an aqueous solution of imidazole with a mole fraction (χ) of 0.1 recorded by Raman spectroscopy (bottom trace) and Infrared spectroscopy in the ATR mode (top trace), showing the 800–1800 cm^–1^ range. The molecular structure and atom labeling of neutral imidazole and its protonated form, imidazolium, are also shown.

**Table 1:** Experimentally Observed Vibrational Modes in Aqueous Imidazole (*χ* = 0.1) and Proposed Assignment According to the Work in Reference 35 Adapted with Permission from Reference 35 ^a^

Observed vibration (ATR) IR	Observed vibration Raman	Assignment based on ref 35
915 (vw)	915 (vw)	58δR_2_–26δR_1_
930 (vw)	930 (vw)	59δR_1_ + 19δR_2_
1065 (s)	1067 (w)	44C_5_N_1_–8N_3_C_4_ + 9δC_5_H
1096 (w)	1098 (w)	41N_1_C_2_–13C_4_C_5_ + 21N_3_C_4_ + 11δNH
1137 (vw)	1137 (m)	50N_3_C_4_ + 32C_5_N_1_
1159 (vw)	1162 (m)	25C_4_C_5_ + 19N_1_C_2_–22δC_5_H + 17δC_4_H
1258 (m)	1260 (s)	51C_2_N_3_ + 27δC_2_H
1328 (m)	1329 (s)	21C_2_N_3_ + 10C_5_N_1_–19δC_5_H–37δC_4_H–25δC_2_H
1429 (m)	1430 (m)	22C_4_C_5_ + 16N_1_C_2_–9N_3_C_4_–41δNH–20δC_4_H
1486 (m)	1489 (m)	16C_4_C_5_ + 13C_2_N_3_–8N_1_C_2_+32δC_5_H–29δC_2_H
1533 (m)	1535 (m)	24C_4_C_5_–13C_2_N_3_ + 32δNH–15δC_4_H
1637 (s)	—	δOH
—	3124 (vw)	13C_4_H–85C_2_H
3131 (vw)	3127 (vw)	53C_5_H–35C_4_H– 12C_2_H
3155 (vw)	3146 (vw)	44C_5_H + 15C_4_H

a Copyright [1993]­[ELSEVIER]. Abbreviations are as follows: s (strong), m (medium), w (weak), vw (very weak), *δ* (deformation mode), and R (ring deformation).

The high frequency region of both Raman and infrared spectra is shown in Figure 2, revealing a stark difference between the strong infrared intensity of the O–H stretching modes of water (ATR IR trace) and the higher sensitivity of Raman to the C–H stretching modes in imidazole. Altogether, these observations provide the basis for relying primarily on infrared spectroscopy in the search for changes as a function of composition from the perspective of the water molecules and, *vice versa*, on the use of Raman spectroscopy in the interest of studying the effect of composition from the perspective of imidazole.

**Figure 2:**
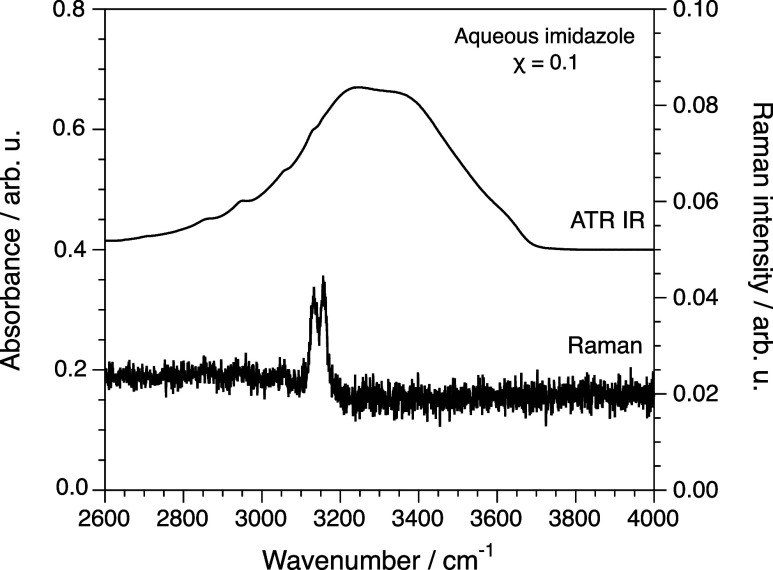
High frequency region of the vibrational spectrum of an aqueous solution of imidazole (χ = 0.1), recorded by Raman and infrared spectroscopy.

### 3.1 Water-Imidazole Interactions and Bond Lengths

Vibrational spectra have been recorded for a series of aqueous imidazole solutions, varying the mole fraction of imidazole, χ, from 0.0 to 1.0. Since previous studies have reported the concentration of these solutions in different ways, a correlation curve has been constructed between the mole fraction of imidazole (χ) and the molarity (M) of solutions, Figure 3. This plot helps the reader compare the results and observations made here with those already reported in the literature.^19,21,22^ Imidazole is highly soluble in water and has a solubility limit of 633 g/L, which corresponds to a ∼9 M solution.

**Figure 3:**
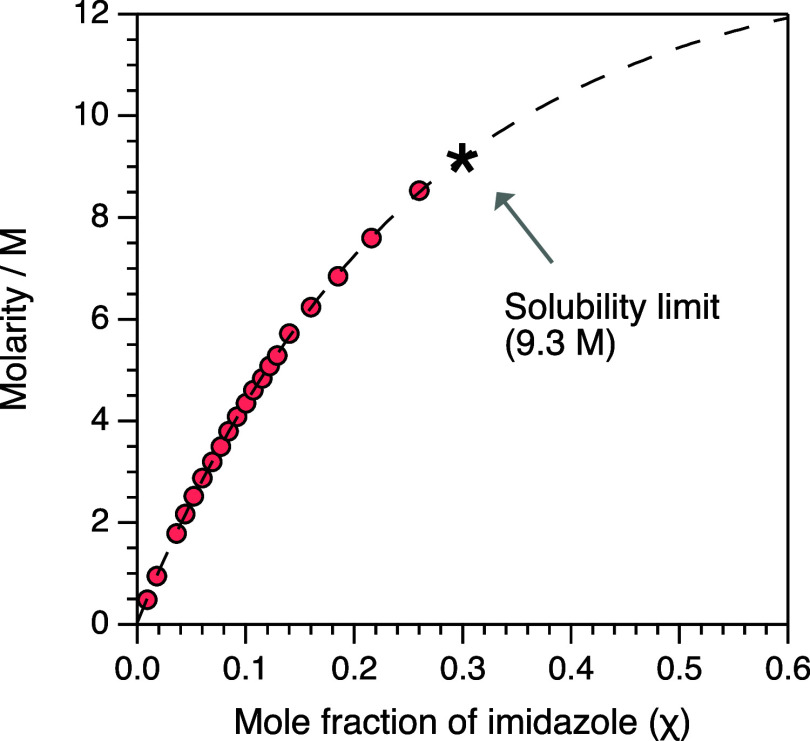
Correlation plot between the mole fraction of imidazole (χ) in an aqueous solution and its molarity (M). This plot has been created using the values reported in Table 2 of ref 19 (Reproduced from ref 19. Copyright [2019] American Chemical Society). The dashed black line serves as a guide to the eye.

The Raman spectra recorded for this series of imidazole solutions are shown in Figure 4, with a vertical offset for better visualization. From top to bottom, spectra are shown for an increasing relative amount of water. A first observation is that the spectra of pure imidazole (χ = 1.0) and of the solution with χ = 0.6 are equivalent, indicating that the local structure of crystalline imidazole is unaffected by the small amount of added water.[Fn fn4] On the other hand, the spectra of solutions with 0.4 ≤ χ ≤ 0.1 display some differences from the spectrum of pure imidazole, remaining similar to each other. The similarity between Raman spectra recorded for aqueous imidazole of different concentrations was observed by M. Thomason,^38^ although no in-depth frequency or line width analysis was performed.

**Figure 4:**
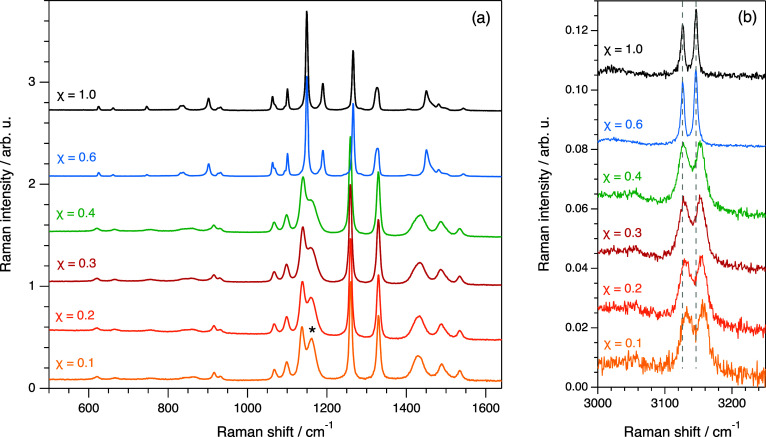
Raman spectra, in the frequency ranges 560–1640 cm^–1^ (a) and 3000–3245 cm^–1^ (b), of aqueous imidazole for molar fractions of imidazole equal to 0.1, 0.2, 0.3, 0.4, 0.6, and 1.0. These spectra are shown with a vertical offset for clarity and after subtraction of the Raman spectrum collected for neat, bulk water (all liquid samples were contained in sealed NMR tubes).

Distinct changes are observed between the Raman spectrum of the solution with χ = 0.6 and the one with χ = 0.4, Figure 4a. Specifically, the mode at 1063 cm^–1^ blue shifts to 1067 cm^–1^, while its closest mode at 1100 cm^–1^ red shifts to 1098 cm^–1^; concomitantly, the modes at 1149 and 1190 cm^–1^ both red shift to 1139 and 1159 cm^–1^ showing a considerable broadening. Further, the mode at 1265 cm^–1^ red shifts to 1260 cm^–1^, while the mode peaked at 1450 cm^–1^ red shifts to 1430 cm^–1^ and broadens. In the higher frequency range of the Raman spectrum, Figure 4b, a blue shift is observed for the two modes, in particular the second one that shifts from 3146 to 3151 cm^–1^.[Fn fn5] The one at 3151 cm^–1^ is a normal mode, assigned to in phase C^4,5^–H stretching. The other modes are of more complex assignment since they include a combination of stretching and sometimes also bending (Table 1). Nevertheless, it is a general rule that a red shift for a stretching mode reflects an elongation of the underlying chemical bond, and *vice versa* for a blue shift. Additionally, a blue shift for a bending mode typically reflects motions around a smaller angle.

It is interesting to note that the transition from the solution with χ = 0.6 to that with χ = 0.4 (equivalent to the highly concentrated solutions discussed in references 19, 21, and 23) corresponds to the addition of one water molecule per imidazole. At this composition, a fundamental structural change is introduced; that is, the direct interaction between one hydrogen of water with the N^3^ atom of imidazole, as evidenced in independent studies based on DFT calculations^18,20^ and supersonic jet infrared spectroscopy.^29^ Hence, the distinct and abrupt spectral changes discussed above directly reflect the new interaction between imidazole and the first water molecule added.

The modes that change the most once this interaction sets in, are the one at 1159 cm^–1^ that shifts by about 30 cm^–1^ and broadens from 5.6 to 35 cm^–1^ in FWHM (an asterisk marks the new position of this mode), and the three modes between 1400 and 1550 cm^–1^, the one at 1430 shifting by 20 cm^–1^ while all three showing a considerable broadening. All these modes include a combination of in phase C^4^–C^5^ with N^1^–C^2^ and/or C^2^–N^3^ stretching, along with C–H and N–H bending. The considerable broadening observed for these modes in the solution with χ = 0.4 is rationalized by the added water molecule breaking the intrinsic structure of crystalline imidazole, allowing for wider angle ranges during N–H and C–H bending. This higher degree of freedom is expected to also come with a red shift. Overall, the red shift of the modes at 1139, 1159, and 1430 cm^–1^ is also congruent with the longer C^4^–C^5^, N^1^–C^2^, N^3^–C^4^ and N^1^–C^5^ bonds observed by X-ray Raman scattering for aqueous imidazole compared to solid imidazole.^23^ In that study, it was also revealed that the N^1^–H bond shortens in aqueous solutions, compared to the case of solid imidazole. The spectroscopic trends revealed by Figure 4 seem also to align with the finding that despite the formation of strong and short OH···N^3^ hydrogen bonds (1.93–1.84 Å),^20^ the covalent bonds involving the N^1^ atom (e.g., N^1^–C^2^ and N^1^–C^5^) are the most affected upon the addition of water.^23^ Table S1 summarizes covalent and hydrogen bond lengths in imidazole, imidazolium, and aqueous imidazole, as reported in two previous studies.^23,35^

By a detailed peak fit procedure, the position and width of key vibrational modes were analyzed upon further dilution, i.e., as a function of decreasing χ. An example of the applied peak fit is shown in Figure S2, while the results obtained from fitting both Raman and infrared spectra are shown in the multiplots of Figures S3 and S4. Previous studies have already established that additional water molecules hydrogen bond to each other, forming a closed chain around imidazole, eventually involving the N^1^H site with water acting as a hydrogen bond acceptor. The latter becomes evident for χ < 0.25.^18,20^ In this configuration, the water molecules proximate to N^3^ are more dispersed in space (being located both above and below the plane of the imidazole ring) and the OH···N^3^ hydrogen bond is significantly shorter (stronger) than the N^1^H···O one, which is also more linear (see also Table S1).^19–21,23,24,36^

Our peak fit results show that the pair of modes observed at ∼1139 and ∼1159 cm^–1^ depend on dilution differently. Upon increased water content, the 1159 cm^–1^ mode blue shifts and narrows, while the mode at 1139 cm^–1^ red shifts but does not change in line width, Figure 5. This red shift is confirmed by infrared spectra, Figure 6a, and unambiguously reflects the elongation of N^3^–C^4^ and C^5^–N^1^ bonds, a trend also observed upon protonation of imidazole.^35^ On the other hand, the blue shift upon dilution of the mode at ∼1159 cm^–1^ is not observed in the infrared spectra, Figure 6b. One possible explanation for this difference is the higher sensitivity of Raman spectroscopy to homonuclear bonds, hence reflecting mostly the fate of the C^4^–C^5^ bond, and the higher sensitivity of infrared spectroscopy to heteronuclear bonds and bending modes, hence revealing mainly the fate of the N^1^–C^2^ and C^4,5^–H bonds. Under this hypothesis, Figure 6b would tell that upon dilution of imidazole in water, the C^4^–C^5^ bond shortens while the same can not be concluded for N^1^–C^2^. Both these bonds are expected to shorten upon protonation (Table S1).^35^ [Fn fn6] The narrowing observed for the 1159 cm^–1^ mode upon dilution is counterintuitive, and yet not rationalized. However, an older investigation of the vibrational modes of imidazole proposes the mode at 1159 cm^–1^ to primarily include N^1^–H bending,^33^ which could narrow as a result of more stable and linear hydrogen bonds upon the formation of closed water chains. Further, the continuous blue shift of the mode at 3151 cm^–1^ (Figure 4b) reveals shorter C^4^–H and C^5^–H bonds, which in turn reflect the experience of weaker hydrogen bonds at these sites in aqueous imidazole compared to solid imidazole. This red shift is congruent with the shorter C^5^–H bond observed upon protonation (Table S1).

**Figure 5:**
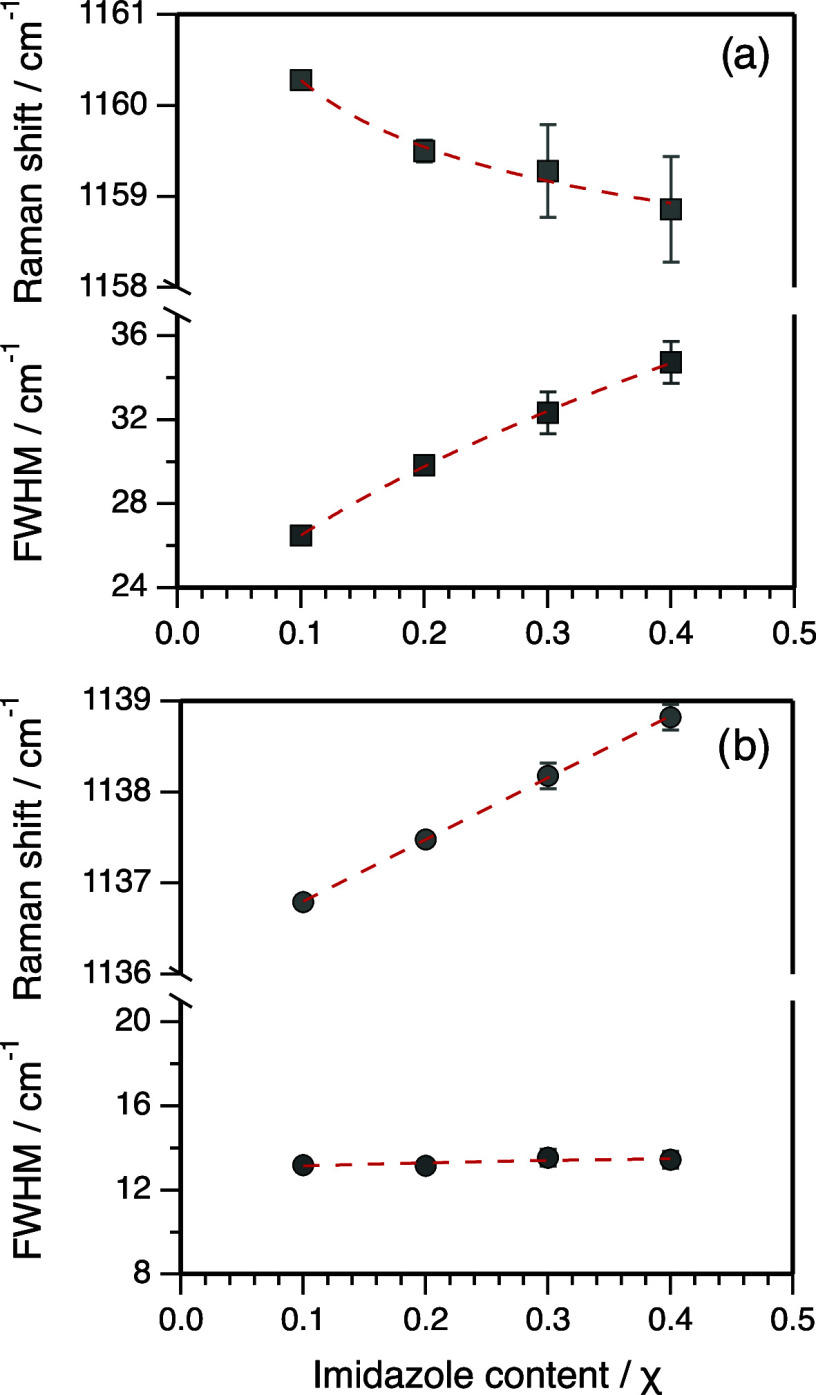
Raman shift (top traces) and FWHM (bottom traces) as a function of composition for the mode at ∼1159 cm^–1^ (a) and ∼1137 cm^–1^ (b), extracted from the peak fitting analysis. Dashed lines are fits to the data points.

**Figure 6:**
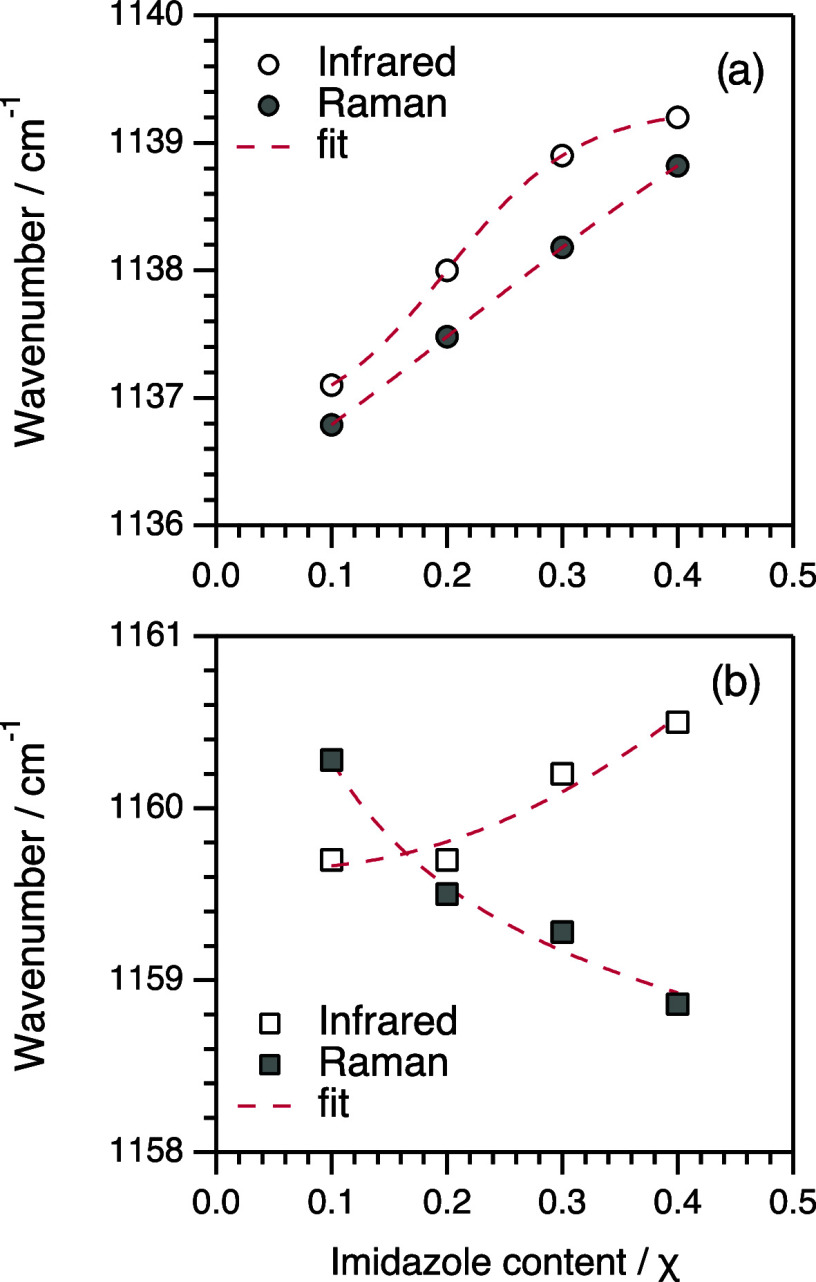
Position of the vibrational modes observed at ∼1137 cm^–1^ (a) and at ∼1159 cm^–1^ (b) by Raman (closed symbols) and infrared (open symbols) spectroscopy. Dashed lines are fits to the data points.

### 3.2 Strong H-Bonds to a Kosmotropic Solute

The high frequency range of the infrared spectra is shown in Figure 7, for solutions of aqueous imidazole as well as for samples of pure water and pure imidazole. In this range, the O–H stretching modes appear, which are very sensitive to the nature of hydrogen bonds formed. The N–H stretching mode of imidazole is seen as a very weak signature at 3124 cm^–1^. The broad feature assigned to the O–H stretching modes has contributions from water molecules hydrogen bonded in different ways. Generally, the lower-frequency part of this broader band is attributed to more strongly hydrogen bonded water, while the higher-frequency part is associated with free or more weakly bonded water molecules. A distinction between symmetric and antisymmetric vibrations can also be invoked in this discussion.^40^ [Fn fn7] A quick inspection of the recorded spectra suggests that the contribution of the components above ∼3300 cm^–1^ decreases with respect to those below this value, for increasing amounts of imidazole; an observation that was further confirmed by a peak fit analysis (Figure S5). This trend reflects a shift to an OH population with more of the hydrogen bond acceptor (A) type. A previous study of water/imidazole complexes by infrared spectroscopy revealed that the O–H stretching modes are mainly affected by the formation of N^1^H···O bonds,^31^ in which water is a hydrogen bond acceptor.

**Figure 7:**
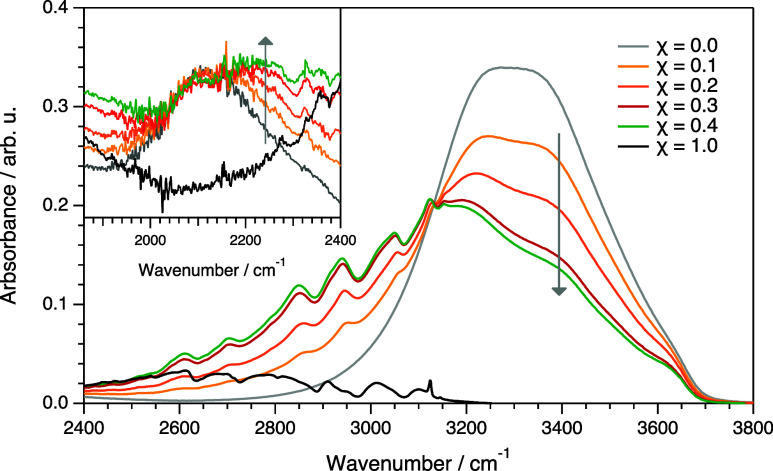
Infrared spectra in the O–H stretching region of neat imidazole, neat water, and aqueous imidazole for χ between 0.1 and 0.4. The inset shows the spectral range of librational modes. In both plots, arrows indicate increasing imidazole content.

The midfrequency region between 1900 and 2500 cm^–1^ of the infrared spectrum of water has been largely overlooked, despite being an important indicator of structure and dynamics in liquid water. The mode observed at 2130 cm^–1^ in pure water is a combination band of H–O–H bending (1637 cm^–1^) and librational (500–700 cm^–1^) modes; librational modes being hindered rotations of water molecules within the liquid structure. This combination mode is sensitive to the rigidity of the H-bonding network;^41^ more precisely, in the presence of solutes, it may shift and hence tell on the kosmotropic (water structure maker) or chaotropic (water structure breaker) nature of the added molecules. In general, a red shift of this combination mode indicates water-solute interactions weaker than water–water interactions, and *vice versa* for a blue shift. To the best of our knowledge, these trends have not been analyzed before for aqueous imidazole. The infrared spectra reported in the inset of Figure 7 reveal a new component peaked at about 2240 cm^–1^,[Fn fn8] which increases with the concentration of imidazole. This evidences the formation of water-imidazole interactions stronger than those between water molecules, hence revealing that imidazole is a kosmotropic solute. This is congruent with the hydrogen bond lengths between imidazole and water (Table S1) being on average shorter than the widely accepted value of 1.97 Å for liquid water. It has previously been shown that the combination mode of water shifts to higher frequencies upon decreased temperature; hence, the blue-shifted mode that we observe upon the addition of imidazole can be attributed to the formation of stronger H-bonds,[Fn fn9] in full agreement with the results retained from the high frequency range of the same infrared spectra (where the O–H stretching modes appear).

### 3.3 Protons in Exchange and Molecular Dynamics

Further information on the nature of water-imidazole interactions has been obtained from the recorded 1D ^1^H NMR spectra, Figure 8. These NMR spectra reveal distinct peaks for the aromatic hydrogen atoms at positions C^2^H and C^4,5^H in imidazole, while the resonances of the N^1^H and OH hydrogen atoms are merged into one signal, indicating proton exchange events between water and imidazole. More precisely, the signal recorded at 4.7 ppm for neat water becomes a merged peak in the solutions, which moves downfield with an increasing concentration of imidazole, accompanied by line broadening (Figure 8a). Opposite, the resonances of the hydrogen atoms bound to the aromatic carbons slightly shift downfield upon dilution in water (Figure 8b). In order to estimate the chemical shift of the N^1^H proton, which is hidden under the merged peak, the population averaged relation under the condition of protons in exchange was used.[Fn fn10] This reveals that the estimated resonance of the hydrogen atoms residing on N^1^ is in the 11–12 ppm range (Figure 8c), in full agreement with the results presented by Teterin et al.^42^ for imidazole diluted in different polar solvents.[Fn fn11] Interestingly, while the resonance of the N^1^H proton in the solid state of imidazole was calculated to be at 15.7 ppm,^43^ it has been observed in the range 13–15 ppm for immobile and strongly hydrogen bonded protons and at ca. 11–12 ppm for mobile proton species.^44^ Hence, through this shift upfield, NMR spectroscopy also reveals that, although the first water molecule affects the environment of the exchangeable proton in the direction of hydrogen bonds weaker than in solid imidazole, further dilution does not further shift the N^1^H resonance significantly. Based on the trends reported by Teterin et al.,^42^ the chemical shift range 11–12 ppm observed in our solutions corresponds to more than 75% of imidazole protons in exchange, while our line width analysis tells a rate of exchange varying from ∼100 s^–1^ (χ = 0.1) to ∼260 s^–1^ (χ = 0.4).[Fn fn12]

**Figure 8:**
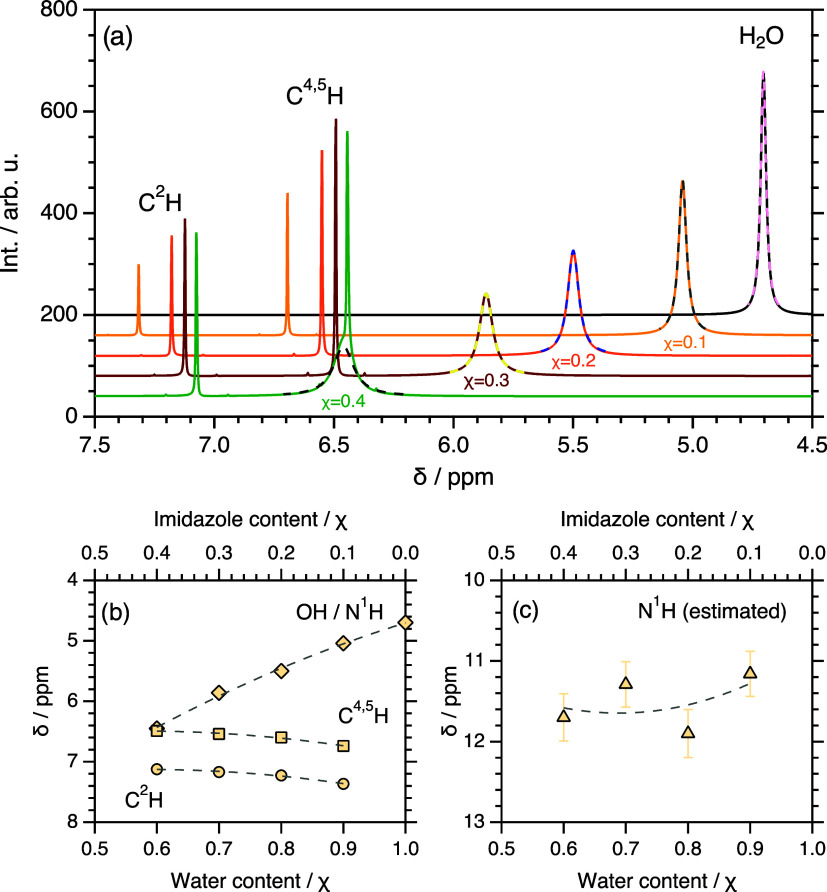
^1^H NMR spectra recorded for the different aqueous imidazole solutions in the ppm range 7.5–4.5. Long-dashed lines are Lorentzian fits (a). Dependence on the water content of the chemical shift for the hydrogen resonances at positions C^2^H and C^4,5^H in imidazole as well as for the merged OH/NH resonance (b). Dependence on the water content of the chemical shift for the hydrogen resonance at positions N^1^H in imidazole (estimated from a population averaged merged resonance) (c). Dashed lines in (b, c) are guides to the eye.

Despite the hypothetical value of aqueous imidazole as a proton conductor, there is little experimental work reported on the transport properties of this solution.^14^ Nevertheless, in the scope of understanding the nature of intermolecular interactions by means of MD simulations, Liem et al.^21^ have computed self-diffusion coefficients for aqueous solutions of imidazole of varying concentrations. In that work, two different sets of self-diffusion coefficients were revealed for water and imidazole, depending on whether the simulations were based on the QCT or the AMBER potential.[Fn fn13] Although both theories predicted higher self-diffusion coefficients for the water molecules than for imidazole in their mixtures, the use of the AMBER potential simulated values almost three times higher than those simulated using QCT. Notably, that work suffered from the lack of experimental data to validate these results, which are provided in this study.

Figure 9 compares, in a single plot, the self-diffusion coefficients computed by Liem et al.^21^ with those experimentally measured by us through PFG NMR measurements. The experimental values measured here agree very well with those computed for water and imidazole in aqueous imidazole based on the QCT potential, demonstrating that it is the model that best represents the real solutions of aqueous imidazole. Simulations based on QCT also reproduced very well, and better than AMBER, the experimental density values.^21^ The better agreement between experimental results and QCT based simulations implies that aqueous imidazole is found primarily in chain-like structures, while stacked dimers are less frequent.^19,21^ In addition, a decrease in diffusivity was observed in the more concentrated solutions, which is simply attributed to increased viscosity.

**Figure 9:**
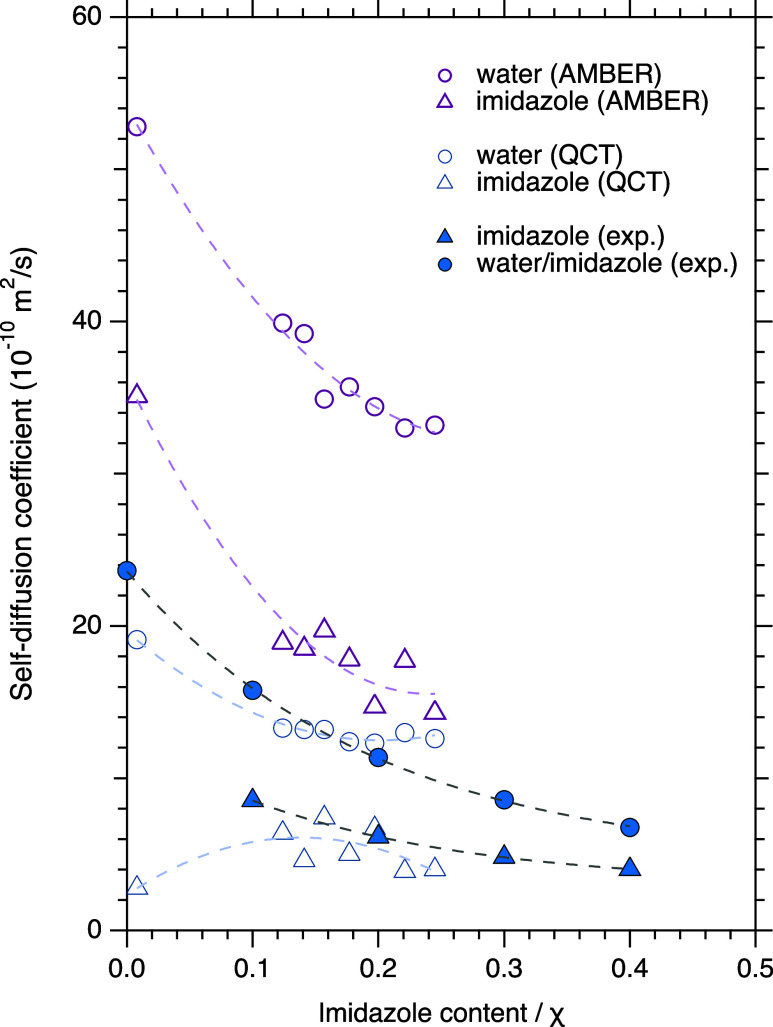
Self-diffusion coefficients estimated from PFG NMR measurements performed at 298 K, using ^1^H resonances distinctively assigned to imidazole (C^2^H, filled triangles) and water/imidazole (OH/NH, filled circles). Values of self-diffusion coefficients obtained for imidazole (open triangles) and water (open circles) from QCT-based (blue) and AMBER-based (purple) simulations by Liem et al.^21^ are also plotted for direct comparison (Adapted from ref.^21^ Copyright [2011] American Chemical Society).

Studies focused on benchmarking the results from MD simulations to data directly achieved from experimental methods are, unfortunately, rare. The data set summarized in Figure 9 represents such a case, revealing that only one of two force fields makes striking contact to experimental results. Some previous works had highlighted this dilemma, that is the very high sensitivity to the choice of force field of dynamic observables. This scientific challenge has been illustrated for molecular conformations and their lifetimes in model systems based on peptides and proteins.^46,47^ The superior accuracy of the QCT potential in describing real, complex systems has been demonstrated for the cases of liquid imidazole and liquid water (of high relevance to this work), in particular by implementation of multipolar electrostatic potentials.^25–27^

### 3.4 Aqueous Imidazole in an Acidic Polymer

In Figure 10, the Raman spectrum of a hydrated perfluorosulfonic acid (PFSA) membrane is reported (mid trace), with its distinct and characteristic features at 804, 970, and 1058 cm^–1^, assigned respectively to the C–S stretching, the C–O–C symmetric stretching and the SO_3_^–^ symmetric stretching of the side chain.[Fn fn14] The corresponding spectrum of a PFSA membrane hydrated with a solution of water and imidazole (with χ = 0.1, top trace) shows the same spectral features, except for the mode at 1058 cm^–1^ being red-shifted to 1054 cm^–1^. Such a red shift has been observed previously as an effect of increased hydration^48,49^ and hence indicates a higher degree of proton dissociation from the −SO_3_H group. This is a desirable situation for achieving good proton conduction in a PFSA membrane and is most likely the result of the aqueous imidazole solution being more basic than pure water. However, in the spectral range 1200–1450 cm^–1^ the signatures of imidazolium are not detected, suggesting that the H^+^ dissociated from the sulfuric acid of PFSA does not protonate imidazole to form imidazolium but is instead bound to water in the form of excess proton complexes. This is opposite to the situation reported by us for a solid salt hydrate, in which the H^+^ dissociated from the strong acid preferred to bind to imidazole over water.^13^ Finally, as simple as it sounds, the advantage of swelling a PFSA with aqueous rather than pure imidazole, which also comes with a higher basicity, is its liquid state.

**Figure 10:**
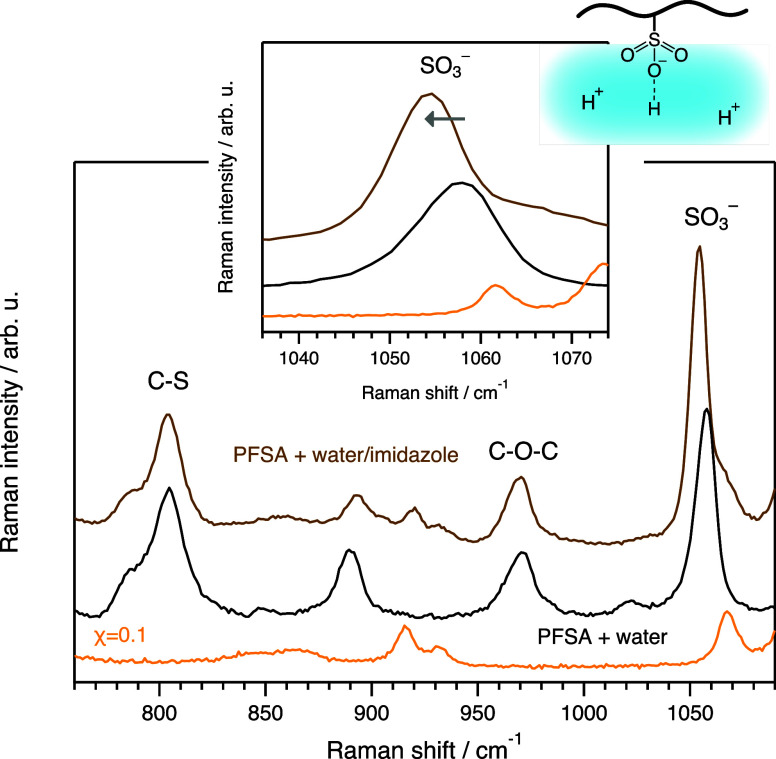
Raman spectra of a hydrated PFSA membrane (black, mid trace), a PFSA membrane hydrated with a mixture of water and imidazole (χ = 0.1, brown, top trace) and a neat water/imidazole solution (χ = 0.1, yellow, bottom trace).

### 3.5 Phase Behavior

The melting temperature of the different compositions of aqueous imidazole was investigated by differential scanning calorimetry (DSC). The recorded DSC traces shown in Figure 11 reveal an interesting phase behavior, with single melting peaks for neat water and imidazole, and more complex curves for the mixtures. The melting peak of pure imidazole is observed at 90 °C and is outside the plotted temperature range. The first endothermic peak, associated with the breaking of hydrogen bonds between water molecules, is shifted to below 0 °C in aqueous imidazole, remaining stable across the different compositions. This is in line with previous findings that imidazole causes minimal disruption to the surrounding network of water molecules.

**Figure 11:**
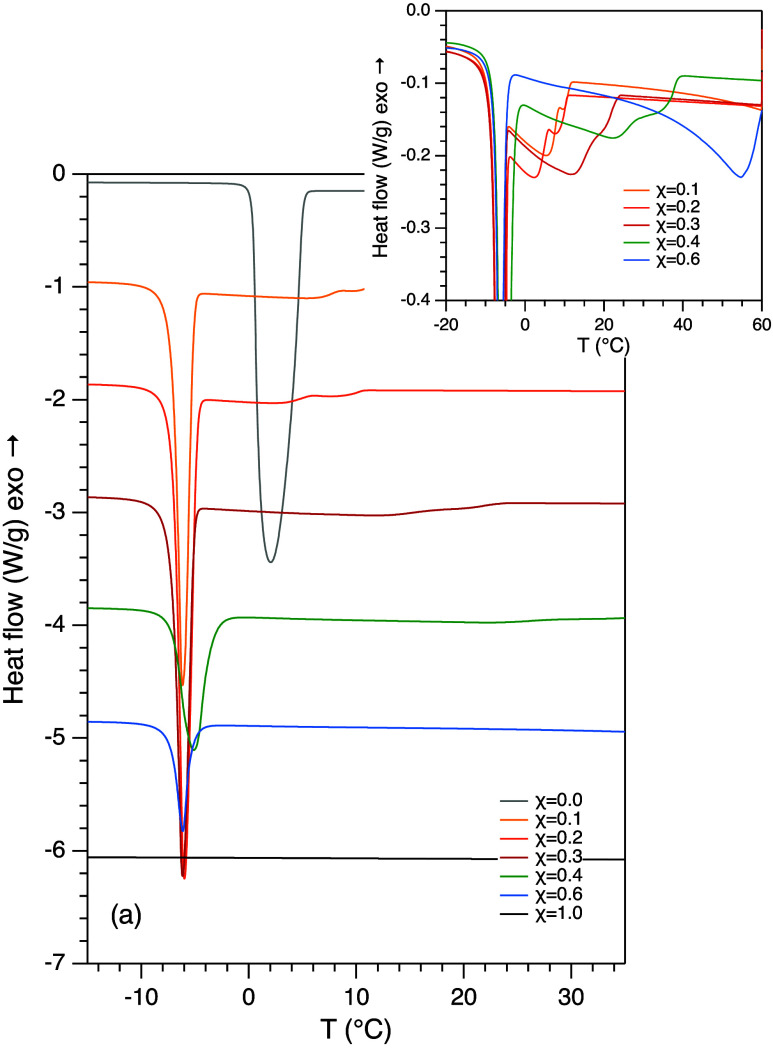
DSC curves of the aqueous imidazole solutions with the mole fraction of imidazole, χ, varying from 0 to 1.0. The inset plot is a close-up of the second endothermic peak.

A second endothermic peak, neither associated with pure water nor pure imidazole, is observed in all solutions, with minima at varying temperatures (see inset). This peak is attributed to hydrogen bonds involving water and imidazole and, by being broad and including multiple components, reflects some structural disorder. This may for example come from the coexistence of linear, stacked, and T-shaped dimers of imidazole.^19,21^ The position of this second peak and its systematic shift to higher temperatures as imidazole content increases (Figure S6) reflect stronger hydrogen bonds than in neat water, in agreement with the spectroscopic results. The persistence of this second peak through all compositions may confirm the finding that, counterintuitively, imidazole-water associations are favored even with increasing imidazole content.^19^

Overall, the results from the experiments reported here converge in telling that, when mixed, water and imidazole interact closely, as revealed by measurable changes in wavenumber and line width (Raman and IR) and protons being in exchange (NMR). These changes are marginal upon further dilution (i.e., beyond the 1:1 water:imidazole ratio), in agreement with predictions from earlier computational studies. The shape of DSC peaks suggests the coexistence of energetically close molecular configurations, although a quantitative estimation of the different types of dimers could not be made. Nevertheless, from a purely vibrational spectroscopic viewpoint, the following qualitative observations can be made. When crossing the 1:1 molar ratio (from χ = 0.6 to χ = 0.4) water disrupts the pristine crystalline structure of imidazole, as seen by the broadening of the peaks that reflects an overall increase of disorder. This broadening is more pronounced for modes that include significant contribution from C–H and N–H bending (Figure S7), which are known to take part in hydrogen bonds. Further, the concomitant blue shift of out-of-plane bending modes (e.g., from 838 to 862 cm^–1^) and red shift of in-plane bending modes (e.g., from 1190 to 1159 cm^–1^ and 1450 to 1430 cm^–1^) is a classic divergent shift that results from hydrogen bonding. These observations may thus reflect the increased population of linear configurations over stacking upon addition of water.

## 4 Conclusions

By presenting results from a number of different spectroscopic techniques (including Raman, infrared and NMR), this work serves as a valuable complement to the previous knowledge of the properties of aqueous imidazole, here with a focus on both intermolecular interactions and mobility, i.e., on structure and dynamics.

Raman spectroscopy reveals abrupt changes in peak position and width that mark the transition from crystalline imidazole to hydrated clusters of imidazole. These changes set in at a 1:1 ratio of water to imidazole and are observed in the spectra of solutions with χ ≤ 0.4. Upon further dilution in water, the hydration structure around imidazole changes marginally, as shown by detectable but small frequency shifts of key vibrational modes.

Upon further dilution, most of the vibrational modes probed by Raman and infrared spectroscopy shift in a direction that reflect the elongation of N^3^–C^4^ and C^5^–N^1^ bonds as well as the shortening of C^4^–C^5^, C^5^–H and, possibly, the N^1^–C^2^ bonds. These are the same trends observed upon protonation of imidazole, suggesting that the strong N^3^···HO hydrogen bond along with the closure of water chains with N^1^H···OH bonds induces a charge delocalization in the aromatic ring congruent with being the precursor for proton transfer. Notably, changes in the hydrogen bond network tending to symmetrization, were also observed for imidazole under pressure.^39^

Infrared spectra in the range of librational modes reveal that imidazole acts as a kosmotropic solute in water, forming strong imidazole-water interactions. The persistence of these interactions across the different compositions is confirmed by DSC, in the form of a second endothermic melting peak.

Importantly, by providing experimental values for self-diffusion coefficients, this work enables discrimination between two computational models used in previous studies,^21^ showing that the QCT model is a good descriptor of aqueous imidazole, while the AMBER approach fails in reproducing measurable properties such as molecular diffusivity. In turn, this indicates that the debated imidazole self-association occurs primarily by linear chains, while H-π stacked and T-shaped dimers are less frequent.

Finally, Raman spectra of a hydrated acidic polymer show that aqueous imidazole is a better solvent for the acidic proton of the −SO_3_H group than neat water. This finding holds promise for using aqueous imidazole as a swelling liquid to support proton conduction in both polymers and alternative solid matrices such as covalent organic frameworks (COFs).

## Supplementary Material



## Footnotes

1 By virtue of being amphoteric molecules, water and imidazole enable proton transport by structural diffusion (a process broader than the Grotthuss mechanism^1,2^ ), by which protons can move faster than their parent molecules through dynamic hydrogen bonds.

2 Note that improved QCT force fields were successfully proved for liquid water and liquid imidazole,^25–27^ providing a basis for the subsequent study of aqueous imidazole solutions^21^ (QCT: Quantum Chemical Topology).

3 With infrared intensities being typically higher for polar and heteronuclear bonds, and Raman intensities being typically higher for covalent and mononuclear bonds. Also, the change in polarizability (Raman) or in dipole moment (infrared) selectively contributes to the recorded relative intensities.

4 Note that the solution with χ = 0.6 corresponds to less than one water molecule per imidazole and should fall beyond the solubility limit.

5 Concomitant, distinct changes are also observed in the wavenumber range 500–1000 cm^–1^, where ring deformation modes appear (Figure S1). For example, the modes at 831/838 and 902 cm^–1^ (assigned to C–H wagging and ring deformation modes, respectively)^39^ blue shift and broaden upon addition of the first water molecule.

6 Diluted imidazole, with water molecules forming the closed chain and interacting with both N^3^ and N^1^H, has a configuration with symmetrical N sites (small Δip) and can be seen as the precursor of protonated imidazole.

7 More sophisticated models use combinations of hydrogen bond acceptor (A) and donor (D) types of OH groups in water, see e.g., Figure 5 in ref 40.

8 The blue shift for this new component is almost entirely attributed to the librational mode, since the bending mode at 1637 cm^–1^ does not change more than 3 cm^–1^ within the composition range analyzed.

9 It is an established knowledge that H-bonds in water weaken upon increased temperatures.

10 The relation used is the following, δ^obs^ = χ^w^·δ^w^ + χ^im^·δ^im^, with χ being the fraction of hydrogen atoms residing on each molecule, and the up-scripts w and im indicating water and imidazole, respectively.

11 These values are the result of N^1^H protons in exchange involved in hydrogen bonds that may change with composition.

12 Calculated as FWHM·800 MHz·π (see ref 45).

13 QCT: quantum chemical topology; AMBER: Assisted Model Building with Energy Refinement.

14 The strong mode at 731 cm^–1^, assigned to the CF_2_ symmetric stretching of the backbone, is outside the shown spectral window.

## Notes

Table with relevant bond lengths and figures complementing the results discussed in the main manuscript, particularly related to vibrational spectra (PDF)
