# Supplementary material for: Local Interactions and Dynamics in Aqueous Imidazole Probed by Vibrational and NMR Spectroscopy
Source: J Phys Chem B. 2026 Jul 20;130(30):7656–66. doi: 10.1021/acs.jpcb.6c02320 (PMC13430631; doi:10.1021/acs.jpcb.6c02320)
Supplement: Supplementary file 1 [file jp6c02320_si_001.pdf]

Supporting Information: Local Interactions and Dynamics in Aqueous Imidazole Probed by Vibrational and NMR Spectroscopy

Nicole Abdou, Eva Dahlqvist, and Anna Martinelli*

Department of Chemistry and Chemical Engineering, Chalmers University of Technology, SE-412 96 Gothenburg, Sweden

E-mail: anna.martinelli@chalmers.se

This document contains figures and plots with information complementary to that already given in the main text.

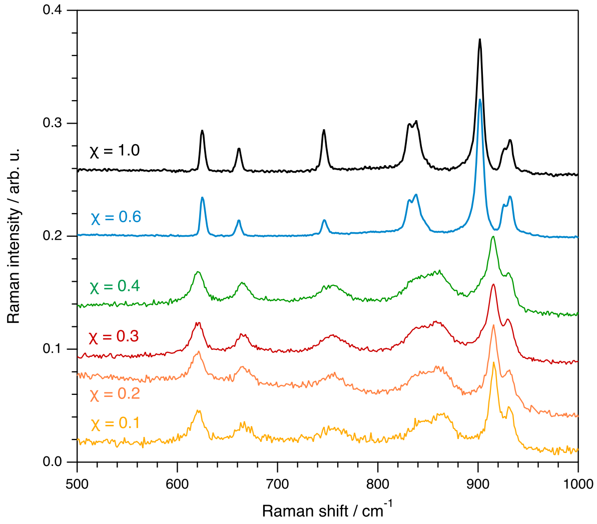

Figure S1: Raman spectra of aqueous imidazole at different concentrations, in the low frequency range.

Table S1: Bond length values (Å) obtained from *ab initio* calculations by Majoube *et al.* (ref. [35]) for neutral imidazole (Im) and its protonated form imidazolium (Im^+) (Adapted with permission from reference [35]. Copyright [1993][ELSEVIER]). Bond length values (Å) obtained from X-Ray Raman spectra by Al-Madhagi *et al.* (ref. [23]) for solid and aqueous imidazole (Reproduced from reference [23]. Available under a CC-BY 3.0 license. Copyright 2018 L.H. Al-Madhagi *et al.*). Relative changes in bond lengths, Δ (%), have been calculated by us from these previously published data.

Chemical bonds	Im (Å)	Im^+ (Å)	Δ (%)	Solid imidazole (Å)	Aqueous imidazole (Å)	Δ (%)
Covalent bond						
N^1-H	0.9946	1.0020	+0.74	1.038	1.014	-2.3
N^3-H	- - -	1.0020	- - -			
C^2-H	1.0635	1.0641	+0.06			
C^4-H	1.0624	1.0624	+0.00			
C^5-H	1.0630	1.0624	–0.06			
N^1-C^2	1.3680	1.3221	-3.30	1.337	1.356	+1.4
C^2-N^3	1.3000	1.3221	+1.70	1.316	1.315	-0.1
N^3-C^4	1.3943	1.3979	+0.26	1.368	1.375	+0.5
N^1-C^5	1.3850	1.3979	+0.93	1.362	1.375	+0.9
C^4-C^5	1.3527	1.3405	–0.90	1.358	1.368	+0.7
Hydrogen bond						
$N^1H \cdots N^3$				1.828		
$OH \cdots N^3$					1.896	
$N^1H \cdots O$					1.945	

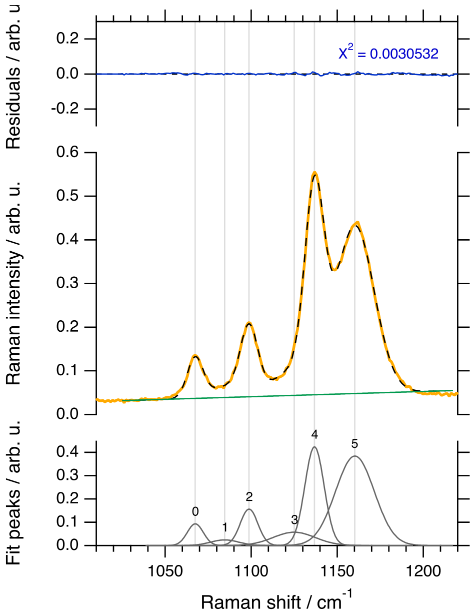

Figure S2: Peak fit procedure and result for the case of the Raman spectrum of aqueous imidazole with $\chi = 0.1$.

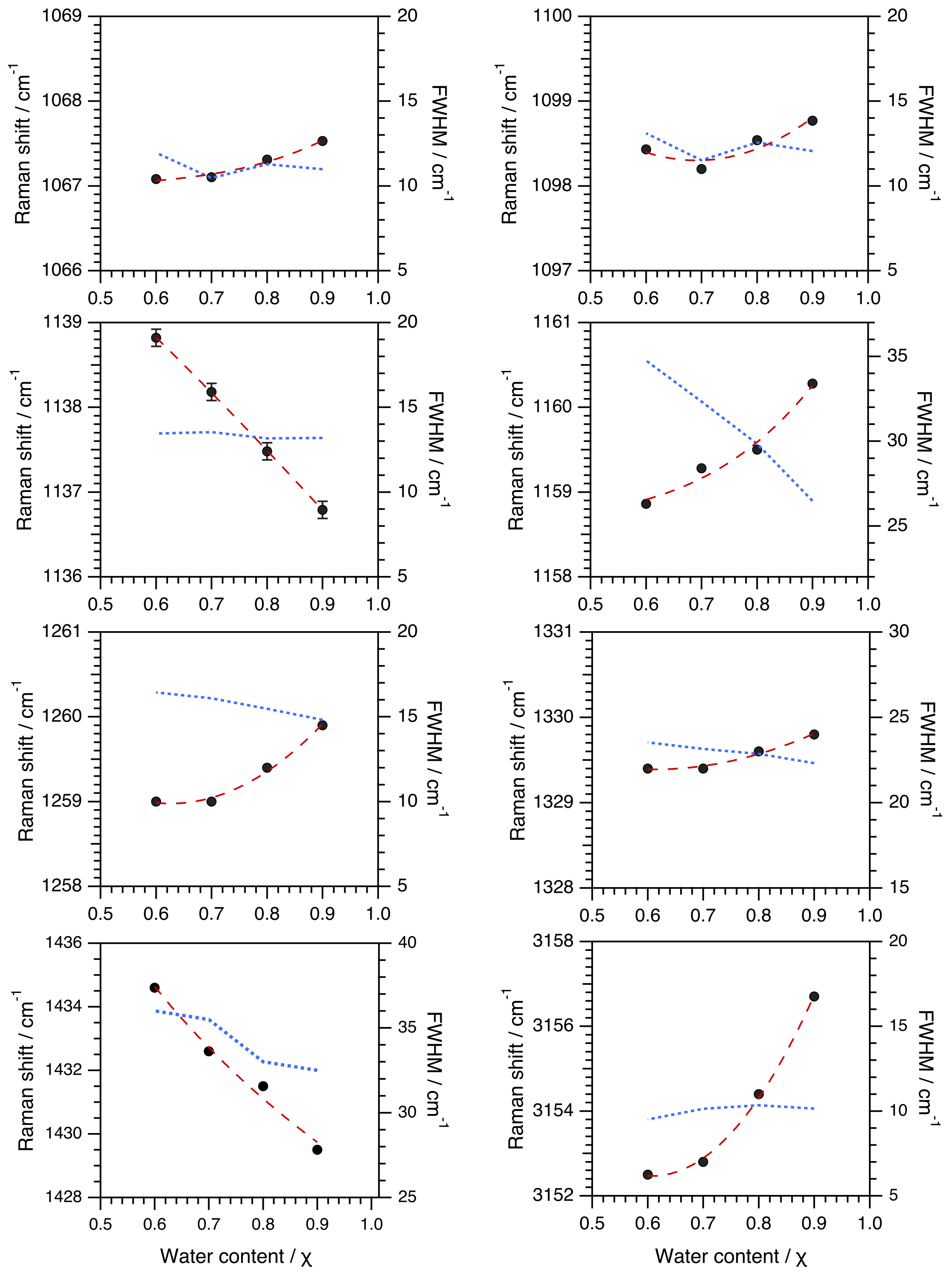

Figure S3: Results from the peak fit analysis of Raman spectra, showing peak positions as black symbols and peak widths as blue dashed lines. For a fair comparison, all left y-axes are 3 cm^{-1} wide, except for the plot showing the peaks at ca 1432 and ca 3155 cm^{-1}, whose y-axis had to be wider. The right y-axes, related to the width of the peaks, is 15 cm^{-1} wide in all cases.

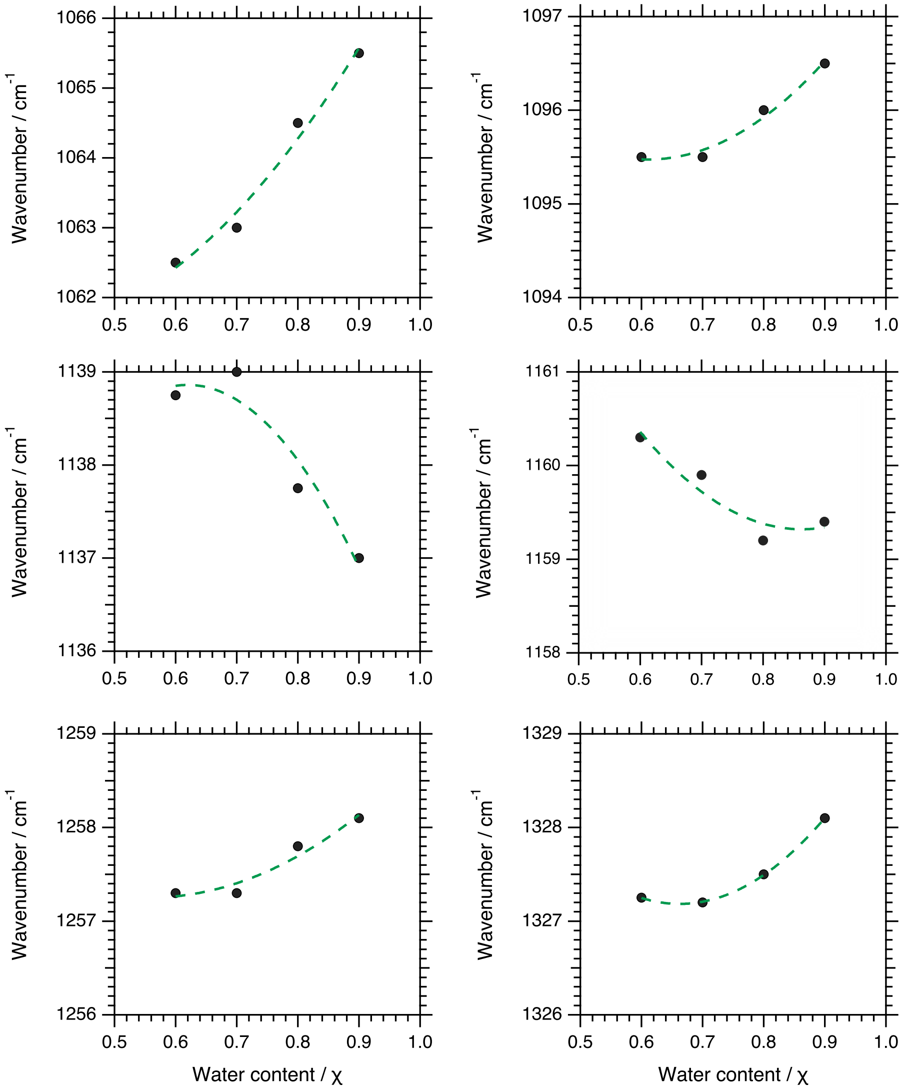

Figure S4: Results from the peak fit analysis of infrared spectra, showing peak positions as black symbols and fitting curves as green dashed lines. For a fair comparison, all left y-axes are 3 cm^{-1} wide, except for the plot showing the peak at ca 1064 cm^{-1}, whose y-axis is 4 cm^{-1} wide.

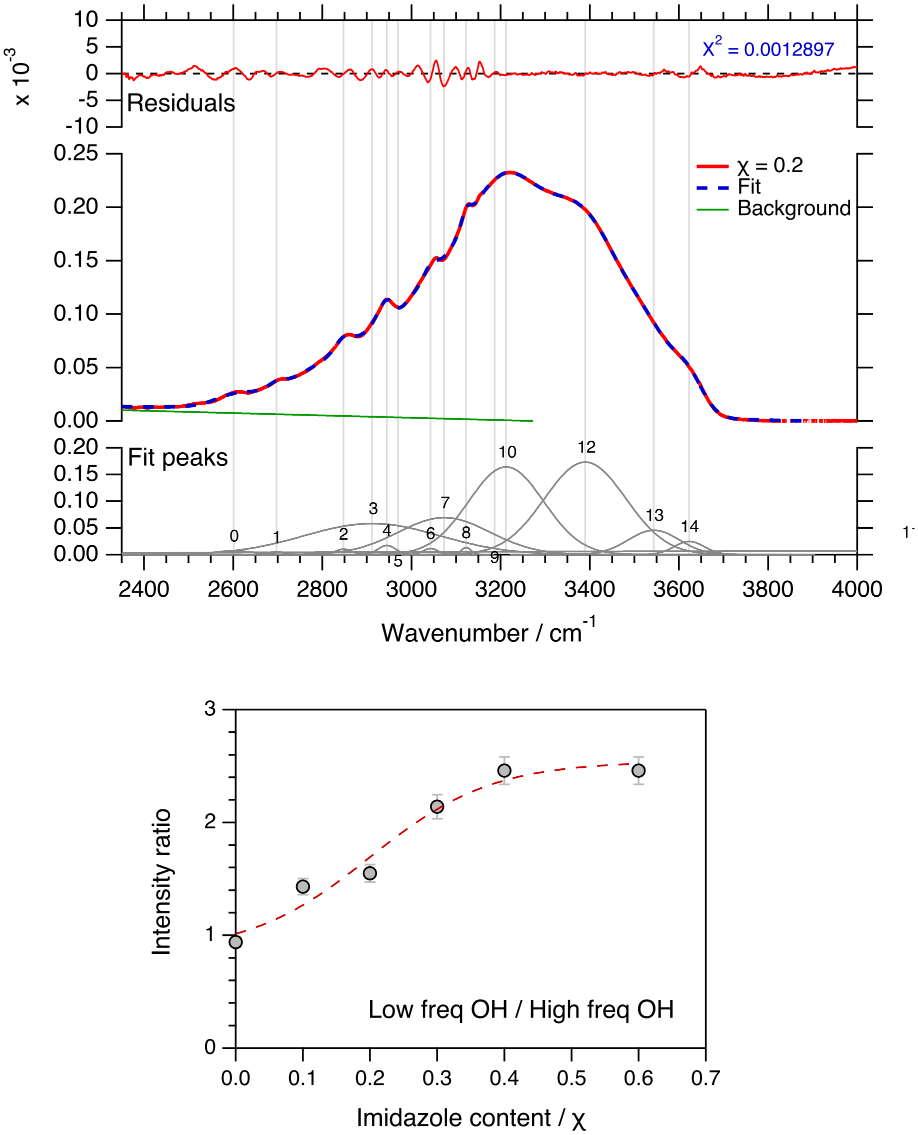

Figure S5: Peak fit procedure and result for the case of the Infrared spectrum of aqueous imidazole with χ=0.2. The bottom plot shows the ratio between the areas under the fitting peaks 10 and 12.

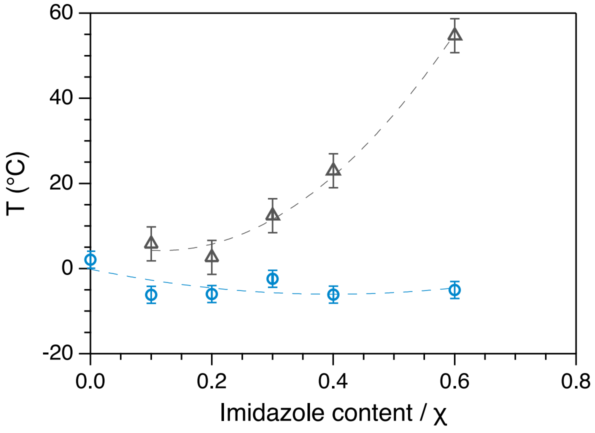

Figure S6: Melting points extracted from the DSC curves for the first (blue) and the second (black) endothermic peak.

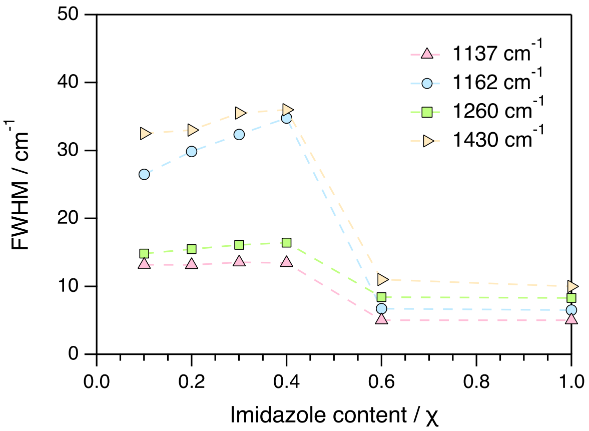

Figure S7: Full width at half maximum (FWHM) of selected peaks observed in the Raman spectra.
